# Vascular Function and Handgrip Strength in Rheumatoid Arthritis Patients

**DOI:** 10.1100/2012/580863

**Published:** 2012-03-12

**Authors:** Mahmoud A. Alomari, Esraa F. Keewan, Rania A. Shammaa, Khaldoon Alawneh, Said Y. Khatib, Michael A. Welsch

**Affiliations:** ^1^Division of Physical Therapy, Department of Allied Medical Sciences, Jordan University of Science and Technology, Irbid 22110, Jordan; ^2^Department of Physiology, Faculty of Medicine, Jordan University of Science and Technology, Irbid 22110, Jordan; ^3^Department of Applied Biology, Faculty of Science and Arts, Jordan University of Science and Technology, Irbid 22110, Jordan; ^4^Department of Internal Medicine, Faculty of Medicine, Jordan University of Science and Technology, Irbid 22110, Jordan; ^5^Division of Rheumatology, Department of Medicine, King Abdulla Hospital, Irbid 22110, Jordan; ^6^Department of Kinesiology, Louisiana State University, Baton Rouge, LA 70803, USA

## Abstract

*Objective*. To examine the relationship of handgrip strength with forearm blood flow (BF) and vascular resistance (VR) in rheumatoid arthritis (RA) patients. *Methods*. Forearm BF at rest (RBF) and after upper arm occlusion (RHBF), and handgrip strength were examined in 78 individuals (RA = 42 and controls (CT) = 36). Subsequently, VR at rest (RVR) and after occlusion (RHVR) were calculated. *Results*. The patients' RBF (*P* = 0.02) and RHBF (*P* = 0.0001) were less, whereas RVR (*P* = 0.002) and RHVR (*P* = 0.0001) were greater as compared to the CTs. Similarly, handgrip strength was lower in the RAs (*P* = 0.0001). Finally, handgrip strength was directly associated with RBF (*r* = 0.43; *P* = 0.0001), and RHBF (*r* = 0.5; *P* = 0.0001), and inversely related to RVR (*r* = −0.3; *P* = 0.009) and RHVR (*r* = −0.3; *P* = 0.007). *Conclusion*. The present study uniquely identifies an association between regional measures of forearm blood flow and handgrip strength in patients and healthy control. In addition, this study confirms the presence of vascular and muscle dysfunction in patients with rheumatoid arthritis, as evidenced by lower forearm blood flow indices, at rest and following occlusion, and lower handgrip strength as compared to healthy individuals.

## 1. Introduction

Rheumatoid arthritis (RA) results in synovium inflammation and long-term joint damage, that can lead to symptoms of chronic fatigue and muscle pain and weakness [1–3]. Subsequently, the patients usually suffer from disability and ultimately reduced quality of life [4]. However, since this inflammation is systemic, the vasculature also undergoes a variety of structural and functional maladaptations [5–9]. In fact, the risk of cardiovascular disease (CVD) for people with rheumatoid arthritis is comparable to those with type 2 diabetes and coronary artery disease and is the major cause of excess mortality in these patients [10–13].

Previous work has shown a strong link between vascular and muscle functions in a variety of populations [14–16]. For example, reduced vascular function was coupled with significantly lower muscular strength in college age and older individuals [14]. Similarly, patients with diabetes mellitus [15] and heart failure [16] have significantly lower vascular and muscular functions compared to age-matched controls.

Despite the potential cardiovascular benefits of exercise for RA patients, as outlined recently [17–19], no studies have examined the relationship between arterial and muscle functions in these patients. It is critical to understand the link between regional vascular and muscular functions, as treatment strategies should aim to alleviate both problems to ultimately maximize physical function and independency. Consequently, the objectives of the current study are to examine the differences in regional arterial function (defined by forearm blood flow before and following a period of upper arm occlusion) and muscle strength (defined by handgrip strength) between individuals with and without RA. A second aim of the study is to examine the relationship between forearm blood flow and handgrip strength. Given previous findings [14–16], it is hypothesized that measures of arterial and muscle functions will be lower in patients with RA than age-matched controls. It is further hypothesized that measures of regional arterial and muscle functions will be directly associated.

## 2. Materials and Methods

### 2.1. Design and Subjects

The study is cross-sectional designed to examine regional vascular and muscular functions in randomly selected RA patients and healthy controls (CTs). The RAs were recruited from the rheumatology out-patient clinic in King Abdullah University Hospital (KAUH), Irbid, Jordan. All patients fulfilled the 1987 American College of Rheumatology Criteria [20] and were diagnosed by a consultant rheumatologist. Apparently, healthy individuals were recruited from the local community and served as the CTs for this study.

Individuals with hypertension, unstable myocardial ischemia, angina, diabetes mellitus, anemia, chronic lung diseases, hypercholesterolemia, renal failure, overly obese, and with major cardiovascular risk factors and smokers were excluded from the study. The patients continue to take prescribed medications. The participants received oral and written information about the study and signed an informed consent forms approved by the Institutional Research Board. Data collection was performed by the same examiner throughout a six-month study period.

### 2.2. Maximum Handgrip Strength

The participants' handgrip strength was measured using a hydraulic hand dynamometer (Asimow Engineering Co., Los Angeles, Calif, USA). The average of 3 consecutive all-out gripping trials was used as the maximum effort. During the measurement, the participant remained upright but slightly bent forward at the waist and the head was in midposition facing straight ahead. The elbow was 90° flexed while the shoulder and wrist were 0° extended. The grip size was adjusted so that the middle finger's midportion (2nd phalanx) was approximately at a right angle [21].

### 2.3. Forearm Blood Flow Assessments

Forearm blood flow was obtained in the dominant forearm using mercury in-silastic strain-gauge plethysmography (EC-5R, Hokanson, Belleview, Wash, USA). Measures were obtained at rest and following 5 minutes of arterial occlusion [22]. This noninvasive technique is based on the assumption that alterations of pressures in strategically placed cuffs allow examination of the rate of change of limb volume thought to reflect blood flow. Strain-gauge plethysmography is a widely used technique to measure blood flow and vascular resistance in the extremities since 1905. It has been validated (*r* ^2^ = 0.8–0.9) with other prominent vascular measures and was recently found highly reliable (ICCC = 0.8–0.9) [14, 23].

Prior to the experiment, blood pressure cuffs were positioned around the upper arm and wrist, and a mercury-in-silastic strain gauge placed around the forearm approximately 10 cm distal to the olecranon process [14]. Following 15 minutes of supine relaxation, resting blood flow (RBF) was measured at 5 millimeter/second paper speed, immediately after occluding the venous system at 7 mmHg below diastolic blood pressure using the upper arm cuff. Thereafter, reactive hyperemic blood flow (RHBF) was obtained, at 25 millimeter/second paper speed, after 5 minutes of arterial occlusion by inflating the upper arm cuff to 240 mmHg. Immediately before the blood flow measurements, the upper arm cuff was deflated to 7 mmHg below diastolic blood pressure. Before resting and arterial occlusion measurements, hand circulation was occluded for 1 minute by inflating the cuff at the wrist to 240 mmHg [14]. Blood pressure and heart rate were measured at rest and after 5 minutes of arterial occlusion using an automated noninvasive standard auscultatory method.

### 2.4. Forearm Blood Flow Calculations

Best-fit slopes drawn at the change in plethysmography volume graph at rest and after occlusion were used to calculate blood flow. The RBF was calculated by dividing the product of 60 seconds and full paper rang by the horizontal distance needed for the slope to reach the top of plethysmography chart. Whereas, RHBF calculated from 60 seconds multiplied by paper speed divided by horizontal distance needed for the slope to reach vertical distance of 20 mm. Forearm RBF and RHBF were expressed as mL of blood per 100 mL tissue per min (mL·100 mL tissue^−1^·min^−1^). Forearm vascular resistance (VR), at rest (RVR) and after occlusion (RHVR), was then determined as (VR = Mean arterial pressure/Blood flow) and expressed as unit (U).

### 2.5. Statistical Analysis

Independent *t*-tests were used to compare forearm blood flow and handgrip strength measures between RAs and CTs. Additionally, Pearson product moment correlations were used to evaluate the relationships between handgrip strength and forearm blood flow measures. All statistical analysis were performed with SPSS 11.0 statistical software using preset alpha at 0.05.

## 3. Results

### 3.1. Participant Characteristics

The baseline characteristics for the 42 RAs and 36 CTs are shown in Table 1. The RAs and CTs had similar age, height, body weight, and body mass index. Table 1 also shows that resting BP and HR were significantly higher in the RA as compared to the CT groups, however, the patients' values were in the normal range. The patients' blood profile was within the normal values, including blood total cholesterol (5.01 ± 1.05 mmol/L), HDL-C (1.32 ± 0.32 mmol/L), LDL-C (3.26 ± 0.76 mmol/L), triglyceride (1.29 ± 0.52 mmol/L), and glucose (4.99 ± 0.76 mmol/L) and similar to those of the CTs. The mean disease duration for the RAs was ~5 years. Erythrocyte sedimentation rate and C-reactive protein averages for the RAs were 63.5 ± 25.7 and 22.3 ± 5.7, respectively. No patients were in an acute phase of their disease throughout the testing procedures. The number of patients taking methroxate was 17, sulfasalazine was 7, prednisolone was 13, infliximab was 2, rituximab was 2, and etanercept was 3. 

Individuals from the greater Irbid area that fit the inclusion and exclusion criteria of the study were recruited for the study as CTs. The CT volunteers filled disease- and medication free questionnaire before accepted into the study.

### 3.2. Maximum Handgrip Strength

**Figure 1:**
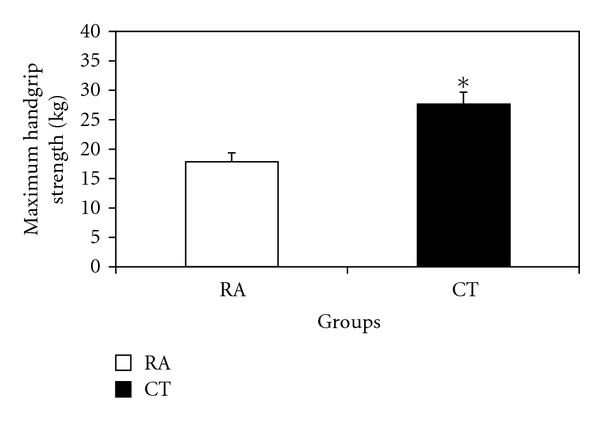
Handgrip strength in RAs and CTs expressed in kg. *P < 0.05 versus CTs.

Handgrip strength was significantly lower (*P* = 0.0001) in the RAs compared to CTs, as depicted in Figure 1.

### 3.3. Forearm Blood Flow Indices

Table 2 reveals that forearm RBF (*P* = 0.02) and RHBF (*P* = 0.0001) were greater in the CTs than in the RAs, whereas RVR (*P* = 0.002) and RHVR (*P* = 0.0001) were significantly lower in the CTs.

### 3.4. Relationships between Handgrip Strength and Forearm Blood Flow Indices

**Figure 2:**
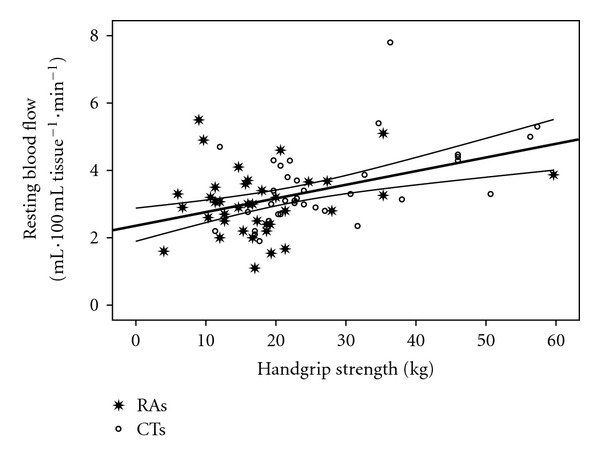
Relationship between handgrip strength and rest forearm blood flow. *r* = 0.43; *P* = 0.0001.

**Figure 3:**
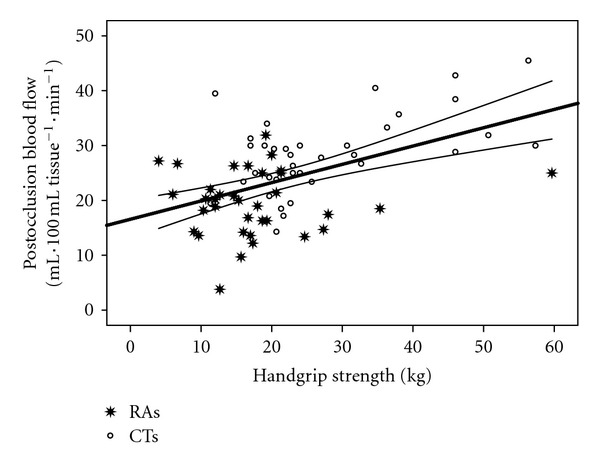
Relationship between handgrip strength and postocclusion forearm blood flow. *r* = 0.5; *P* = 0.0001.

**Figure 4:**
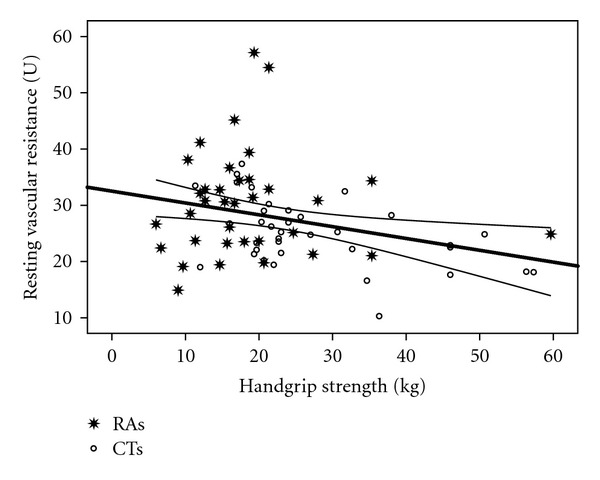
Relationship between handgrip strength and resting vascular resistance. *r* = −0.3; *P* = 0.009.

**Figure 5:**
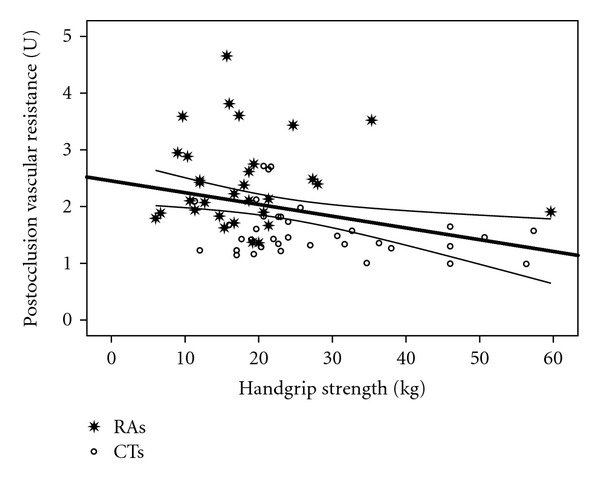
Relationship between handgrip strength and postocclusion vascular resistance. *r* = −0.32; *P* = 0.007.

Figures 2 and 3 show that handgrip strength was significantly correlated with RBF (*r* = 0.43; *P* = 0.0001) and RHBF (*r* = 0.5; *P* = 0.0001). Figures 4 and 5 show that handgrip strength was inversely related to RVR (*r* = −0.3; *P* = 0.009) and RHVR (*r* = −0.3; *P* = 0.007).

## 4. Discussion

The aim of the present study was to examine regional measures of arterial and muscular functions in rheumatoid arthritis patients and healthy controls. The present study uniquely identifies an association between regional measures of forearm blood flow and handgrip strength in RAs and CTs. In addition, this study confirms the presence of vascular and muscular dysfunction in RAs, as evidenced by lower RBF, RHBF, and handgrip strength as compared to CTs.

### 4.1. Handgrip Strength

As indicated in Figure 1, handgrip strength was 30% lower in RAs than in CTs. This is consistent with previous reports indicating 16–29% lower handgrip strength [24, 25] in RA patients. Strength deterioration is well documented in RA reaching up to 75% [3], even apparent in newly diagnosed patients [1]. Importantly, reduction in handgrip and finger pinch strengths were most related to hand disability and articular damage [26].

The mechanism(s) contributing to muscle weakness are not precisely known, however, systemic inflammation seems to lead to joint impairment, characterized by joint swelling, pain, and tenderness [27, 28]. Consequently, chronic joint inflammation leads to damage and deformity, increased physical inactivity, muscle dysfunction, and diminished functional capacity [4]. In addition, many studies have attributed muscle wasting and weakness to hypermetabolic, -catabolic, and -cachectic conditions, subsequent to chronic inflammatory in RA [27, 28]. Evidently, an improvement in the inflammatory index was associated with an improvement in muscle strength in RA patients after 6-month of dynamic strength training [1].

Often due to the nature of the disease, this chronic inflammatory state contributes to a vicious cycle of greater functional impairment and disability. The proposed model indicates that disablement process among those patients begins with pathology/injury or defect resulting in impairment of the tissue, organ, and/or system [4].

### 4.2. Forearm Blood Flow

The lower blood flow and higher vascular resistance at rest and following a period of occlusion, in the current study are consistent with the stated hypothesis. As shown in Table 2, RBF and RHBF were 14 and 31%, lower in the RAs, whereas RVR and RHVR were 20 and 38%, higher in the RAs. These differences fit the growing evidence that patients with RA suffer from vascular dysfunction [6, 7, 9, 29].

Unfortunately the design of the present study does not allow an in depth discussion of the specific maladaptive vascular changes and the underlying causes. However, pro-inflammatory cytokines produced in the synovial fluid, and enter the systemic circulation, can directly alter vascular function by reducing the vascular wall endothelium permeability and increased leukocytes and platelets adhesion [30, 31]. Cytokines can also induce their effect indirectly through their actions on insulin sensitivity, the lipid milieu, nitric oxide bioavailability, fibrinogen, and antioxidant capacity [30, 32]. Cumulatively, these changes shift the balance between proatherogenic and antiatherogenic factors toward greater vulnerability for vascular maladaptations. Overtime, the functional alterations lead to structural remodeling, often defined by larger conduit arteries that are stiffer, less elastic, and thicker. Of course these changes are in part determined by the changes in the tissue (e.g., muscle atrophy), which that part of the vasculature feeds.

### 4.3. Relationships between Maximum Handgrip Strength and Forearm Blood Flow Indices

The unique contributions of this study are presented in Figures 2–5. The figures are consistent with the second stated hypothesis that regional measures of vascular function will be associated with measures of muscle function. This is the first study to report such a relationship in RA patients, which is consistent with previous reports in other populations [14–16, 33]. The findings suggest that alterations in vascular function contribute to a decrease in muscle function in RA patients. Several important observations can be made from the present data. First, as in Figures 2 and 4, resting forearm blood flow is lower, and forearm resistance is higher in the RA patients compared to CTs. The reduced resting forearm blood flow could in part be due to muscle atrophy which is typically seen in RA patients [24, 25]. However, the increased forearm resistance may indicate additional changes that are not solely due to atrophy. In fact an increase in resistance probably indicates there is some degree of vascular remodeling. Consequently it is not a surprise that even the resting flow/resistance measures are associated with handgrip strength. Perhaps more important is the observation that the vascular response to a period of occlusion is related to handgrip strength and quite different between the groups. As seen in Figure 3 it is clear that ~50% of the RA patients are below the bottom CI line and only 17% are above the CI line. In contrast, ~63% of CTs are above the top CI line. In regards to Figure 5, ~45% of the RA patients are above the top CI line and only 20% are below the bottom CI line, whereas 67% of CTs are below the bottom CI line. This provides proof of the concept that vascular function is compromised across the handgrip strength continuum in the RA patients.

The mechanisms that may explain these vascular differences between the groups may include reduced endothelium and metabolic vasodilatation, vasoresponsiveness, and capillary-to-tissue ratio, subsequently blunted blood flow to muscle tissue. Certainly the sequence of these changes remains to be revealed. However, since inflammation and regular physical inactivates seem common denominators for muscular and vascular dysfunctions in RA [4, 27, 28, 30, 32], these changes might be reciprocated and the result of a continuous vicious cycle of muscle and vessel deterioration. Therefore, we think the present observations are unique in a sense that it is the first study to establish a link between vascular and physical function in patients with rheumatoid arthritis. Consequently, contributes to the existing literature in identifying several unique aspects that warrant further discussion and research.

Either way, this regional specific link is an important consideration for intervention. Adding exercise to the treatment regimen might be a valuable complement to manage the complex and interrelated nature of the disease. It is well known that a regional specific exercise training regimen induces significant and rapid localized vascular and muscular changes [34–37]. Given localized exercise stimulus is less stressful on the whole body, regional-specific exercises may provide an option for maintaining vascular and muscular functions. Subsequently, preserving physical function, especially in patients where systemic inflammation is a serious concern. We look forward to presenting such data in the future.

## 5. Conclusion

The present study uniquely identifies an association between regional measures of forearm blood flow and handgrip strength in rheumatoid arthritis patients and healthy controls. In addition, this study confirms the presence of vascular and muscle dysfunction in patients with rheumatoid arthritis, as evidenced by lower forearm blood flow indices, at rest and following occlusion, and lower handgrip strength as compared to the controls. The associations between regional measures of vascular and muscle functions suggests a strong possibility that regional-specific training may preserve functionality in patients with rheumatoid arthritis.

## Figures and Tables

**Table 1:** Participant characteristics.

	Patients (*n* = 42)	Control (*n* = 36)	*P* value
Females/males	37/5	31/5	
Age (years)	34.0 ± 10.3	30.4 ± 10.0	0.13
Height (cm)	162.0 ± 7.6	165.0 ± 10.7	0.27
Body weight (kg)	70.8 ± 14.1	70.1 ± 14.7	0.84
Body mass index (kg/m^2^)	26.7 ± 5.4	26.2 ± 5.0	0.56
Resting heart rate (b/min)	82.0 ± 9.1	74.14 ± 10.1	0.001
Resting systolic pressure (mmHg)	120.7 ± 15.3	113.6 ± 13.0	0.040
Resting diastolic pressure (mmHg)	76.6 ± 11.5	69.44 ± 8.0	0.003
Resting mean arterial pressure (mmHg)	91.3 ± 12.1	84.2 ± 8.9	0.005

Data presented as mean ± SD.

**Table 2:** Forearm arterial indices.

	Patients (*n* = 42)	Control (*n* = 36)	*P* value
Arterial inflow (mL/100 mL/min):			
Rest	3.0 ± 1.0	3.5 ± 1.2	0.02
Postocclusion	19.7 ± 6.1	28.5 ± 7.1	0.0001

Vascular resistance (U):			
Rest	31.2 ± 9.7	25.0 ± 6.0	0.002
Postocclusion	5.0 ± 1.8	3.1 ± 0.9	0.0001

Data presented as mean ± SD.
